# Heritability of dietary food intake patterns

**DOI:** 10.1007/s00592-012-0387-0

**Published:** 2012-03-15

**Authors:** Linda van den Berg, Peter Henneman, Ko Willems van Dijk, Henriette A. Delemarre-van de Waal, Ben A. Oostra, Cornelia M. van Duijn, A. Cecile J. W. Janssens

**Affiliations:** 1 Department of Human Genetics, Leiden University Medical Center, Leiden, The Netherlands; 2 Department of Pediatrics, Leiden University Medical Center, Leiden, The Netherlands; 3 Department of General Internal Medicine, Leiden University Medical Center, Leiden, The Netherlands; 4 Department of Clinical Genetics, Erasmus University Medical Center, Rotterdam, The Netherlands; 5 Genetic-Epidemiology Unit, Department of Epidemiology and Department of Clinical Genetics, Erasmus University Medical Center, Rotterdam, The Netherlands; 6 Department of Epidemiology, Erasmus University Medical Center, Rotterdam, The Netherlands

**Keywords:** Heritability, BMI, Food intake

## Abstract

The quality and quantity of food intake affect body weight, but little is known about the genetics of such human dietary intake patterns in relation to the genetics of BMI. We aimed to estimate the heritability of dietary intake patterns and genetic correlation with BMI in participants of the Erasmus Rucphen Family study. The study included 1,690 individuals (42 % men; age range, 19–92), of whom 41.4 % were overweight and 15.9 % were obese. Self-report questionnaires were used to assess the number of days (0–7) on which participants consumed vegetables, fruit, fruit juice, fish, unhealthy snacks, fastfood, and soft drinks. Principal component analysis was applied to examine the correlations between the questionnaire items and to generate dietary intake pattern scores. Heritability and the shared genetic and shared non-genetic (environmental) correlations were estimated using the family structure of the cohort. Principal component analysis suggested that the questionnaire items could be grouped in a healthy and unhealthy dietary intake pattern, explaining 22 and 18 % of the phenotypic variance, respectively. The dietary intake patterns had a heritability of 0.32 for the healthy and 0.27 for the unhealthy pattern. Genetic correlations between the dietary intake patterns and BMI were not significant, but we found a significant environmental correlation between the unhealthy dietary intake pattern and BMI. Specific dietary intake patterns are associated with the risk of obesity and are heritable traits. The genetic factors that determine specific dietary intake patterns do not significantly overlap with the genetic factors that determine BMI.

**Electronic supplementary material:**

The online version of this article (doi:10.1007/s00592-012-0387-0) contains supplementary material, which is available to authorized users.

## Introduction

Obesity is a major health problem. Results of twin and family studies suggest that 40–80 % of the variation in body mass index (BMI) in humans can be explained by genetic factors [1]. Some of these genetic factors seem to influence eating behavior [2]. Significant genetic contributions have been reported for appetitive traits such as total energy intake, macronutrient intake, food preference, and satiety responsiveness [2–4].

Several studies have suggested that dietary data can be described in terms of a limited number of patterns, corresponding to healthy and unhealthy eating habits [5–7]. Data on heritability estimates for such dietary intake patterns are sparse: only two twin studies are available in the literature. A study by Van den Bree et al*.* [7] identified a healthy and an unhealthy eating pattern in middle-aged and elderly male and female twins in the US. Genetic factors explained approximately one-third of the variation in these patterns. Teucher et al. [6] found five heritable dietary patterns in UK female twins, aged 18–79 years old (fruit and vegetable, high alcohol, traditional English, dieting, low meat), with heritability estimates ranging from 41 to 48 %. Knowledge about the genetic overlap between dietary patterns and BMI is lacking in the literature. However, De Castro et al*.* [8] did report that 44 % of the variance in meal frequency and 65 % of the variance in meal size were attributable to heredity. Insight in the interplay between diet, genes and adiposity is crucial for understanding the pathophysiology of obesity.

In the present study, we used self-report questionnaire data from the Erasmus Rucphen Family (ERF) study. We conducted a survey to determine the frequency of consumption of vegetables, fruit, fruit juice, fish, unhealthy snacks, fast food, and soft drinks among the participants in ERF. In order to determine the total genetic susceptibility underlying quantitative and qualitative food intake, we assessed the heritability estimate of these dietary intake traits. Furthermore, we aim to discover the presence of inter-correlations, that is, genetic and environmental overlap, between the dietary food intake traits and BMI.

## Subjects and methods

### Subjects

Subjects were participants from the Erasmus Rucphen Family (ERF) study. This community was founded in the middle of eighteenth century and includes approximately 3,000 individuals, who were not selected based on health information, but rather comprise living descendants of 22 couples who had at least 6 children baptized in the community church around 1850–1900. Details about the genealogy and the basic genetic structure of this isolate have been described elsewhere [9–11]. All ERF participants underwent extensive medical examinations in the period between 2002 and 2005. Data on, for example cardiometabolic risk parameters [12], migraine [13], and physiological parameters [14] were obtained. In addition, all subjects were invited to fill out questionnaires in May 2006. Details of the questionnaire study were described previously [9]. The study protocol was approved by the medical ethics board of the Erasmus MC Rotterdam, the Netherlands. All investigations were carried out in accordance with the Declaration of Helsinki.

### Data collection

Height and weight were self-reported by participants [9]. The correlation between physician-assessed body weight at the time of medical examinations and self-reported body weight at the time of the questionnaire study was 0.93 [9]. Of the 2,766 participants in the ERF study, 1,713 (62 %) returned the questionnaire. Although non-responders were older, less educated, and had less often had a positive family history of hypertension, there were no major differences between responders and non-responders [9]. Weight and height were used to calculate BMI (weight in kg divided by squared height in m). Underweight was defined as BMI < 18.5 kg/m^2^; normal weight as BMI 18.5–24.99 kg/m^2^; overweight as BMI 25−29.99 kg/m^2^; and obese as BMI ≥ 30 kg/m^2^. The questions about food intake were part of a larger self-report questionnaire [9]. Questions addressed the number of days (0–7) on which the participants consumed vegetables, fruit, fruit juice, fish, unhealthy snacks, fast food, and soft drinks. A translation of the questions can be found in Supplementary Information S1.

### Statistical analysis

Principal components analysis (PCA) and statistical tests were performed using SPSS version 16.0 (IBM Corporation, Somers, NY, USA). The maximum percentage of missing values per questionnaire item was 3.7 %. We used PCA with varimax rotation for factor analysis. The Kaiser criterion was used to determine the number of factors to be extracted. Gender differences in factor scores were tested with independent samples *t* tests. The relationship of age with factor scores was evaluated using linear regression analysis. BMI class differences in factor scores were evaluated using ANCOVA, with age and gender as covariates. Heritability estimates were obtained using SOLAR software, a software package for genetic variance components analysis (version 2.05) (http://solar.sfbrgenetics.org). The polygenic model with covariates gender and age was applied. The polygenic model assumes that an infinite number of genetic factors with a small additive effect contribute to the phenotypic variation of the trait under study. Inbreeding coefficients did not contribute significantly to the heritability estimations, and this covariate was excluded in the reported analysis. Moreover, BMI also did not contribute significantly to the heritability estimations (data not shown) and was therefore excluded in the reported analysis as well. In family-based heritability estimations, the shared familial environment may contribute significantly to the estimate of heritability. Therefore, we estimated this second variance component, the sibship effect (*S*). This effect is an estimate of phenotypic similarity induced in the progeny of parents by the effects of shared environment and genetic dominance effects. To determine the genetic and environmental correlations between traits, bivariate heritability analyses were performed for BMI with all food intake questions as well with the factors that resulted from PCA. Environmental and genetic correlations range from −1.0 to 1.0. They indicate whether the observed correlation between traits is due to genetic factors, environmental factors, or a combination of both. A significant genetic correlation implies that there is a significant correlation between traits caused by common genetic factors; a significant environmental correlation implies that there is a significant correlation between traits caused by the same environmental factors. Combining the environmental and genetic correlations results in a phenotypic correlation that is corrected for family relationships. The principle of bivariate heritability analysis is explained in the papers of DeStefano et al*.* [15] and Aukes et al*.* [16].

## Results

We analyzed questionnaires of 1,690 participants, of whom 3.6 % were underweight, 38.3 % had a normal weight, 41.9 % were overweight, and 16.2 % were obese. Age of the participants ranged from 19 to 92 years, and 42 % were men. Further characteristics of the cohort and mean questionnaire scores are presented in Table 1.

**Table 1:** Characteristics of the study population (*n* = 1,690)

	Women (58 %)	Men (42 %)
Age (year)	51.2 ± 16.4	51.0 ± 15.4*
BMI (kg/m^2^)	25.8 ± 5.0	27.1 ± 4.0**
Dietary intake questionnaire^a^		
Vegetables (d/wk)	4.3 ± 1.5	4.1 ± 1.5**
Uncooked vegetables (d/wk)	2.2 ± 1.5	2.0 ± 1.4
Fruit (d/wk)	5.0 ± 2.3	4.6 ± 2.3**
Juice (d/wk)	2.8 ± 2.5	2.4 ± 2.3**
Fish (d/wk)	1.0 ± 0.7	1.0 ± 0.0
Snack (d/wk)^b^	4.0 ± 2.5	3.4 ± 2.3**
Fast food (d/wk)^b^	0.8 ± 0.8	1.0 ± 0.1
Soft drink (d/wk)	1.9 ± 2.6	3.0 ± 0.1**

Values represent mean ± standard deviation

*Significantly different from women (*P* value <0.05), **(*P* value <0.01)

^a^d/wk: days per week

^b^Examples regarding snacks: chips, French fries, peanuts, cheese, cookies, pastry, chocolate, candy. Examples regarding fast food: ready-to-eat frozen meals such as pizza; McDonalds, Burger King, or fried meals. See also supplementary information S1

Principal components analysis showed that the questionnaire items could be grouped in a healthy and unhealthy dietary pattern, explaining 22 and 18 % of the phenotypic variance, respectively (Table 2). In other words, people who reported to eat vegetables on many days, often also reported to eat fruit, fruit juice, and fish on many days, whereas people who reported to eat unhealthy snacks on many days, often also reported to eat fast food and drink soft drinks on many days. Thus, this implies that the “Healthy” factor score in this study represents a food intake pattern based on frequent consumption of unprocessed high nutrient but low energy density food while the “Unhealthy” factor score represents a food intake pattern involving frequent consumption of processed and low nutrient but energy-dense foods. “Healthy” factor scores of female participants (mean factor score = 0.096) were significantly higher than those of male participants (mean factor score = −0.13), *t* test *P* < 0.001. There was no significant gender difference in scores on the “unhealthy” factor, *t* test *P* = 0.85. Age had a statistically significant effect on factor scores, with older people scoring higher on the “healthy” factor (*P* < 0.001) and lower on the “unhealthy” factor (*P* < 0.001). “Unhealthy” dietary pattern scores significantly differed between normal weight and overweight/obese subjects (*P* = 0.01). Scores on the “healthy” factor did not differ between BMI groups (*P* = 0.27).

**Table 2:** Result of principal components analysis of dietary intake traits

	Factor 1 (“Healthy,” 22 %)	Factor 2 (“Unhealthy,” 18 %)
Vegetables (d/wk)	0.55	−0.20
Uncooked vegetables (d/wk)	0.55	−0.18
Fruit (d/wk)	0.65	−0.08
Juice (d/wk)	0.61	0.33
Fish (d/wk)	0.53	−0.17
Snack (d/wk)^a^	0.02	0.69
Fast food (d/wk)^a^	−0.24	0.62
Soft drink (d/wk)	−0.15	0.61

Results were obtained using varimax rotation with kaiser normalization. In brackets is the percentage of variance explained by the factor. d/wk: days per week. Numbers in the table are factor loadings of the questionnaire items

^a^Examples regarding snacks: chips, French fries, peanuts, cheese, cookies, pastry, chocolate, candy. Examples regarding fast food: ready-to-eat frozen meals such as pizza; McDonalds, Burger King, or fried meals. See also supplementary information S1

Heritability and sibship effect of the individual questionnaire items as well as the “Healthy” and “Unhealthy” factors are reported in Table 3. Heritability estimates for individual food categories ranged from non-significant for fast food to 0.26 for vegetable intake. None of the traits showed a significant sibship effect. The dietary patterns had a heritability of approximately 0.32 for the “healthy” and 0.27 for the “unhealthy” pattern (Table 3). In the present study, the heritability and sibship effect of BMI were estimated to be 0.31 and 0.062, respectively. Bivariate heritability analyses were performed to estimate both genetic and environmental correlations between questionnaire scores and BMI. None of the dietary traits displayed a significant genetic correlation with BMI. The environmental correlation (Rho E) of BMI with the “unhealthy” factor was found to be significant (Rho E = 0.16, *P* = 0.01).

**Table 3:** Heritability of dietary intake traits

Trait	Heritability^a^			Sibship effect		
	*h* ^2^	SE	*P*	*S*	SE	*P*
Vegetables (d/wk)	**0.26**	0.05	7.8 × 10^−09^	0.0	0.0	NA
Uncooked vegetables (d/wk)	**0.13**	0.05	7.2 × 10^−04^	0.0	0.0	NA
Fruit (d/wk)	**0.23**	0.06	3.4 × 10^−05^	0.006	0.05	0.45
Juice (d/wk)	**0.12**	0.05	0.048	0.0	0.0	NA
Fish (d/wk)	**0.12**	0.05	0.001	0.0	0.0	NA
Snack (d/wk)^b^	**0.26**	0.05	2.7 × 10^−09^	0.0	0.0	NA
Fast food (d/wk)^b^	0.004	0.05	0.47	0.04	0.05	0.21
Soft drink (d/wk)	**0.08**	0.05	0.037	0.0	0.0	NA
“Healthy” (factor)	**0.32**	0.06	3.1 × 10^−09^	0.0	0.0	NA
“Unhealthy” (factor)	**0.27**	0.06	3.8 × 10^−06^	0.005	0.05	0.50

*h* ^2^ Heritability, *SE* standard error of heritability of sibship estimate, *S* sibship effect, *NA* not applicable

^a^Heritability estimates were based on a polygenic model including age and gender as covariates. Estimates that differed significantly from zero are listed in bold

^b^Examples regarding snacks: chips, French fries, peanuts, cheese, cookies, pastry, chocolate, candy. Examples regarding fast food: ready-to-eat frozen meals such as pizza; McDonalds, Burger King, or fried meals. See also supplementary information S1

## Discussion

In this study, we examined dietary intake patterns, and we estimated the heritability of the intake patterns. PCA showed that food intake items could be grouped in a healthy and unhealthy dietary intake pattern. We found lower self-reported frequency of consumption of healthy foods in men compared with that of women. Older participants scored higher on the healthy pattern and lower on the unhealthy pattern. The heritability estimate of BMI in the ERF cohort (*N* = 2,506) was earlier reported to be 0.44, without considering the sibship effect [17], which is as high as the estimate for BMI in this cohort, sibship effect consideration included (data not shown). The intake patterns had a significant heritability of approximately 30 %. Our results are very much in line with those of the twin study of Van den Bree et al. [7]. These authors also reported a healthy and unhealthy pattern after factor analysis, with heritability estimates of approximately 33 % for both patterns. Van den Bree et al. also reported healthier intake patterns in women compared with those in men. Healthier eating patterns in older people were previously reported by Teucher et al. [6].

An unhealthy intake pattern was significantly associated with the risk of being overweight or obese in our study. However, we failed to detect a significant genetic correlation between the dietary intake traits and BMI. Instead, the correlation between an unhealthy intake pattern and BMI seems the result of the same environmental factors because the environmental correlation between these traits was significant. We can speculate that certain environmental and/or social economic factors promote unhealthy eating habits, resulting in a high BMI. However, the direction of the relationship cannot be determined in a cross-sectional study, so it is also possible that high BMI results in unhealthy dietary patterns.

Our results indicate that the genetic factors that influence unhealthy or healthy dietary patterns as measured with our questionnaire are different from the genetic factors that determine BMI. Other studies have reported significant genetic correlations between BMI and different types of behavior traits. For instance, heritability estimates ranging from 26 to 63 % have been reported for cognitive restraint, 9–60 % for emotional eating and 45–69 % for uncontrolled eating as measured with the three-factor eating questionnaire [18, 19]. These behavioral traits did show a significant genetic correlation with BMI in the study of Keskitalo et al*.* [19]. Other studies showed a genetic correlation between BMI and total energy intake [20, 21]. It thus seems that genetic factors that influence the above-mentioned traits overlap with those affecting BMI, which is not the case for the dietary intake traits that we analyzed in this paper. Interestingly, our results indicate a significant environmental correlation between dietary intake traits and BMI. Considering the questionnaire used in the present study (supplementary information S1) and those of the other studies, the dietary food intake traits differ substantially. Former reports primarily focus on physiology or energy density, that is, quantitative food intake traits [18–20], while the present study questionnaire however emphasized more on the qualitative food intake, that is, the quantity of the of type of food. The preference for certain types of food could imply detection of the genetic component underlying taste perception like bitterness (PTC or PROP) [26, 27].

Taken together, a possible explanation for these observations is that it is quantity rather than quality of dietary intake that shows a genetic correlation with BMI.

There are a number of methodological issues to this study. First, the present analyses are based on self-report questionnaires rather than on real-life observations. Underreporting of food intake is a potential problem, especially in obese people [22]. Direct observation or weighed food records provide more accurate measures of dietary intake. However, being observed or weighing intake may induce behavioral changes such as a reduced-calorie diet, so these measures may not be valid [23]. In addition, such methods are expensive and thus difficult to apply in large studies. Food frequency questionnaires are considered the most cost-effective tool for assessing usual intake in large population studies [24]. The strengths and weaknesses of various methods of diet assessment were nicely discussed by de Castro [25] and by Barrett-Connor [23]. A second limitation is that our questionnaire has not been validated with other measures such as direct observations of food intake. Third, heritability estimates are by definition limited to the population under study. It is likely that the living habits of ERF participants differ from those of families living in other parts of the Netherlands or in other countries.

In conclusion, we demonstrated that dietary intake patterns are heritable traits that can predict the risk of obesity. However, the genetic factors that determine intake patterns do not significantly overlap with the genetic factors that determine BMI, stressing the complexity of the phenotype BMI.

## Electronic supplementary material

Below is the link to the electronic supplementary material.

Supplementary material 1 (DOCX 16 kb)

## Abbreviations

BMI: Body mass index
SE: Standard error of the mean
PCA: Principal component analysis
ANCOVA: Analysis of covariance
*h*^2^: Heritability
*S*: Sibship effect
RhoG: Genetic correlation
RhoE: Environmental correlation
