# Supplementary material for: Heritability of dietary food intake patterns
Source: Acta Diabetol. 2012 Mar 15;50(5):721–6. doi: 10.1007/s00592-012-0387-0 (PMC3898132; doi:10.1007/s00592-012-0387-0)
Supplement: Supplementary file 1 — Supplementary material 1 (DOCX 16 kb) [file 592_2012_387_MOESM1_ESM.docx]

**Supplementary Information S1:** translation of the questionnaire items

**Eating and eating habits**

The following questions concern your eating habits.

**How many days a week do you usually eat cooked vegetables, fried vegetables, lettuce, or other raw vegetables?**

*Note that vegetables in dishes such as hotchpotch also count, but lettuce on a sandwich does not count.*

|  | 0 | 1 | 2 | 3 | 4 | 5 | 6 | 7 | days |
| --- | --- | --- | --- | --- | --- | --- | --- | --- | --- |
| 1. cooked or fried vegetables |  |  |  |  |  |  |  |  |  |
|  |  |  |  |  |  |  |  |  |  |
| 2. lettuce/raw vegetables |  |  |  |  |  |  |  |  |  |

**How many days a week do you usually eat fruit or drink fruit juice** (fresh or bottled) ?

|  | 0 | 1 | 2 | 3 | 4 | 5 | 6 | 7 | days |
| --- | --- | --- | --- | --- | --- | --- | --- | --- | --- |
| 3. number of days fruit a week |  |  |  |  |  |  |  |  |  |
|  |  |  |  |  |  |  |  |  |  |
| 4. number of days fruit juice a week |  |  |  |  |  |  |  |  |  |

**5. How many days a week do you eat fish?**

|  | 0 | 1 | 2 | 3 | 4 | 5 | 6 | 7 | days |
| --- | --- | --- | --- | --- | --- | --- | --- | --- | --- |
|  |  |  |  |  |  |  |  |  |  |

**6. How many days a week do you eat snacks or other in-betweens?**

Examples are chips, French fries, peanuts, cheese, cookies, pastry, chocolate, candy. Coffee table cookies also count. Fruit and raw vegetables do **not** count.

|  | 0 | 1 | 2 | 3 | 4 | 5 | 6 | 7 | days |
| --- | --- | --- | --- | --- | --- | --- | --- | --- | --- |
|  |  |  |  |  |  |  |  |  |  |

**7. How many days a week do you eat fast food or ready-to-eat meals?**

Examples are ready-to-eat frozen meals such as pizza; McDonalds, Burger King, or fried meals.

|  | 0 | 1 | 2 | 3 | 4 | 5 | 6 | 7 | days |
| --- | --- | --- | --- | --- | --- | --- | --- | --- | --- |
|  |  |  |  |  |  |  |  |  |  |

**8. How many days a week do you drink soft drinks or energy drinks?**

Examples are Coca cola, Pepsi, Fanta, AA and Aquarius.

|  | 0 | 1 | 2 | 3 | 4 | 5 | 6 | 7 | days |
| --- | --- | --- | --- | --- | --- | --- | --- | --- | --- |
|  |  |  |  |  |  |  |  |  |  |
